# Case Report: Fatal disseminated cytomegalovirus infection following cyclophosphamide therapy in a young patient with systemic lupus erythematosus

**DOI:** 10.3389/fimmu.2025.1610687

**Published:** 2025-10-20

**Authors:** Mohanad Jaber, Loai Muhtasib, Hebatallah Qawasmeh, Mariam Thalji, Hiba Y. Yaghmour, Hasan Hroob, Ashraf Al-Zughayyar, Majed Dweik

**Affiliations:** ^1^ Faculty of Medicine, Palestine Polytechnic University, Hebron, Palestine; ^2^ Medical Intensive Care Unit, Al-Ahli Hospital, Hebron, Palestine; ^3^ Faculty of Medicine, Al-Quds University, Jerusalem, Palestine; ^4^ Radiology Department, Faculty of Medicine, Palestine Polytechnic University, Hebron, Palestine

**Keywords:** cytomegalovirus (CMV), systemic lupus erythematosus (SLE), sepsis, rheumatology, case report, DIC, colitis

## Abstract

Systemic lupus erythematosus (SLE) is an autoimmune disease characterized by autoantibody production and systemic inflammation. Immunosuppressive treatment is often required to achieve remission. While opportunistic infection rates have risen in this patient population, cytomegalovirus is one of the most lethal opportunistic infections with fatal consequences. Herein, we report a case of an 18-year-old female patient with a two-year history of SLE complicated by lupus nephritis who presented with gastrointestinal symptoms while on immunosuppressive medication. She then developed a cascade of serious complications, including colitis, fulminant liver failure, acute pancreatitis, and pneumonitis, which progressed to disseminated intravascular coagulation (DIC). Detailed investigations were conducted, and the patient was diagnosed with disseminated cytomegalovirus infection. Multidisciplinary supportive management failed to save her life. Disseminated CMV infection is a rare but deadly condition in patients with SLE. This case emphasizes the importance of maintaining a high level of suspicion and considering CMV infection as a differential diagnosis in patients with SLE on immunosuppressive therapy who have obvious gastrointestinal symptoms. This allows for early detection, timely management, and administration of antiviral drugs, leading to improved overall patient health outcomes.

## 1 Introduction

Systemic lupus erythematosus (SLE) is an autoimmune disease characterized by the presence of circulating autoantibodies and immune complexes. It can affect multiple vital organs, resulting in a wide range of clinical manifestations. To attain remission and avoid flare-ups, SLE patients frequently require intensive immunosuppressive therapy (1, 2). High-dose corticosteroids with cyclophosphamide, antimetabolite drugs such as methotrexate and azathioprine, and biologic therapies such as belimumab and rituximab are widely used in treatment (1, 3).

Despite the fact that these drugs help reduce morbidity from SLE, such immunomodulation raises the risk of infection with opportunistic pathogens, which are the primary cause of morbidity and mortality in SLE patients (1, 3, 4). CMV is one of the most prevalent opportunistic infections. In rare cases, particularly those with delayed management, CMV can cause serious organ dysfunction, disseminated intravascular coagulation (DIC), and even death. Therefore, screening and prevention protocols for this patient population are necessary (3, 5–7).

In this report, we describe a rare case of an 18-year-old female patient with SLE who developed a CMV infection, which led to fatal consequences, such as colitis, pneumonitis, pancreatitis, hepatitis, and DIC.

## 2 Case presentation

An 18-year-old female was diagnosed with SLE complicated with grade 4 lupus nephritis at the age of 16, based on a kidney biopsy. She received multiple therapies, including high-dose steroids, tacrolimus, and rituximab, 1 year ago, with a good response. Two weeks before admission, she developed a flare-up of lupus nephritis, with rapid deterioration of kidney function. Kidney biopsy was not available at that time; therefore, she was treated in another hospital for RPGN with cyclophosphamide 500 mg once daily for two weeks and showed good improvement in her clinical and laboratory kidney function.

**Figure 1:**
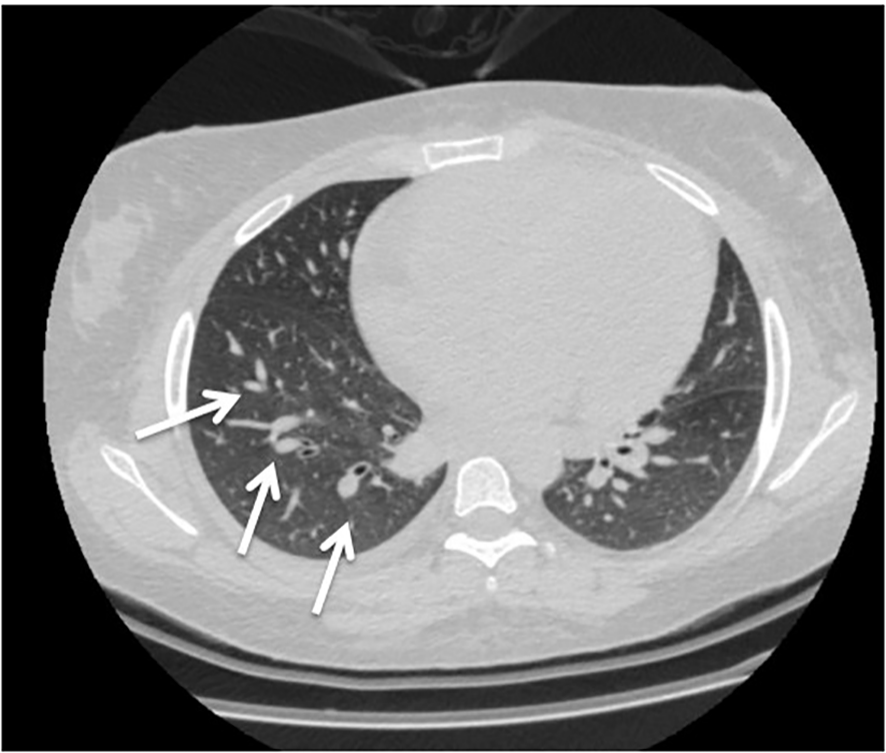
Computed tomography (CT) revealed bilateral mild pleural effusion and nodular consolidation in the lower right lobe encircled by ground-glass opacities (white arrow). She was admitted for additional assessment and treatment.

Two weeks after cyclophosphamide therapy, the patient presented to our department with complaints of watery diarrhea, vomiting, hematochezia, shortness of breath, and general weakness. Upon examination, the patient appeared ill and pale. Her blood pressure was 190/85 mmHg, her heart rate was 85 beats per minute, her oxygen saturation (SpO₂) was 96%, respiratory rate was 19 breaths/min, and temperature was 37.9 °C. A respiratory examination revealed decreased air entry and basal crepitations. Grade 4 bilateral lower limb pitting edema was observed. Results of laboratory tests performed at admission revealed anemia, high neutrophils with low leukocytes, lymphopenia, and elevated creatinine, which was her usual baseline; otherwise, the results were normal (**Table 1**). Hemolytic anemia was confirmed by blood smear examination. Subsequent Computed tomography (CT) revealed bilateral mild pleural effusion and nodular consolidation in the lower right lobe encircled by ground-glass opacities. The patient was admitted for further assessment and treatment. (**Figure 1**). Echocardiography revealed mild left ventricular and left atrial dilatation.

**Table 1:** Laboratory data on admission.

Parameters	Result	Normal range
Hemoglobin	7.1	12-16 g/dl
Hematocrit	20.6%	37-48%
Mean corpuscular volume	85.3	82-92 fL
Mean corpuscular hemoglobin	29.5	27-31 pg
Mean cellular hemoglobin concentration	34.6	32-36 g/dl
Red blood cell count	2.4	4.2-5.4 million cells/mcL
Red cell distribution width	15.1	11.5-13.5%
White blood cells	2.9	5-10 cells/mcL
Neutrophils	85.2	45-65%
Lymphocyte	7.7	25-45%
Platelet	81000	150,000-400,000 cells/dl
Reticulocyte	4.2	0.5-1.5%
Aspartate aminotransaminase	13	0-31 U/L
Alanine aminotransaminase	14	0-45 U/L
Prothrombin time	13.3	11-13.5 seconds
Partial thromboplastin time	29	25-36 seconds
International normalized ratio	1.02	<1
Creatinine	2.3	0.5-1.4 mg/dl
Sodium	142	132-148
Potassium	3.5	3.5-5.7
Chloride	109	96-109
Albumin	3.1	3.5-5.5
C3	12	80-176
C4	<2.9	15-57
C-reactive protein	20	Up to 6mg/l

On the next day, she developed a high-grade fever, her symptoms worsened, and her vital signs became unstable. She was treated with broad-spectrum intravenous antibiotics. A full septic workup was performed including blood, urine, stool, and sputum cultures, nasal swabs, and lumbar puncture, with no growth. Serologic tests for HAV, HCV, and HBV were negative, while anti-CMV IgM and IgG antibodies were positive (**Table 2**). Subsequent Polymerase chain reaction for multiple viruses was conducted. PCR testing was not available and had to be outsourced to external laboratories for confirmation. Late test results were negative for all viruses except CMV, which revealed a high viral load (**Table 3**). Therefore, she was diagnosed with CMV colitis and IV Ganciclovir was initiated. However, her condition rapidly deteriorated and she developed epigastric and right upper quadrant pain. Liver function tests showed a sharp elevation in liver enzymes: aspartate aminotransaminase 7300 (0 U/L–31 U/L) and alanine aminotransaminase 1660 (0 U/L–45 U/L), both amylase with lipase were elevated, amylase 228 (28 U/L–100 U/L), lipase 379 (0 U/L–78 U/L), and International normalized ratio >8 which indicates that she had fulminant hepatic failure and pancreatitis.

**Table 2:** Serological tests.

Lab test	Result	Normal range
CMV Immunoglobulin M (IgM)	>1.3	Negative <0.7 index Broadline 0.7-0.9 index Positive>0.9 index
CMV Immunoglobulin G (IgG)	>250	>6 AU/ml
Hepatitis A Immunoglobulin M (IgM)	0.26 index	Negative<0.8 index Broadline 0.8-1.2 index Positive >1.2 index
Hepatitis C	Negative	Negative <1.0 index Positive >1.0 index
Hepatitis B core	0.06	Negative<1.0 S/CO Positive <1.0 S/CO
Hepatitis B surface antigen	0.2	Negative <1.0 index Positive >1.0 index

**Table 3:** Polymerase chain reaction tests results.

Test	Result
Herpes simplex virus 1,2 (PCR Qualitative)	Negative
Cytomegalovirus (PCR Qualitative)	Positive
Influenza A,B (RT-PCR)	Negative
Avian flu (RT-PCR)	Negative
Enterovirus (RT-PCR)	Negative
Influenza A (HINI) (RT-PCR)	Negative
RSV (RT-PCR)	Negative
Avian influenza A (H7N9)	Negative
Parainfluenza viruses (RT-PCR)	Negative

Following this, she developed severe vaginal bleeding, and a coagulation profile was performed. The results were as follows: D-dimer, 3,805 ng/ml (0 ng/ml–250 ng/ml); fibrinogen, 318 mg/dl (15 mg/dl–350 mg/dl); platelets, 27 × 109/L (150–400 × 109/L); prothrombin time, >90 s (11 s–13.5 s); and partial thromboplastin time, 52.5 s (25 s–36 s). The patient had disseminated intravascular coagulation (DIC), and according to the International Society of Thrombosis and Hemostasis (ISTH) Criteria for DIC, the score was above 5, which is compatible with overt DIC. Management of DIC was initiated with fresh frozen plasma; however, the patient died due to sepsis, DIC, and multi-organ failure (clinical course summarized in **Table 4**).

**Table 4:** Clinical course and management.

Interval	Symptoms/Findings	Diagnostic Decisions	Test Results	Treatments
Day 1	**Symptoms:** She had watery diarrhea, vomiting, hematochezia, shortness of breath, and general weakness. **By examination**: her blood pressure was 190/85, her temperature was 37.9°C, decreased air entry, basal crepitus, and bilateral lower limb edema grade 4.	**Labs:** CBC, LFT and creatinine. **blood smear.** **Imaging:** Subsequent computed tomography **Echocardiography**.	**Labs:**anemia, high neutrophils with low leukocytes, lymphopenia,and elevated creatinine **blood smear:** hemolytic anemia. **Imaging:** bilateral mild pleural effusion and nodular consolidation at the lower right lobe encircled by ground glass opacities. **Echocardiography**: revealed mild left ventricular and left atrium dilatation	The patient was treated as hospital-acquired pneumonia, so the patient was started on: Piperacillin-tazobactam 2.25*4 Levofloxacin 500 E.O.D According to GFR Methylprednisolone 60 mg*1 Famotidine 20 mg ×2 daily Escitalopram 20 mg ×1 daily Rosuvastatin 10 mg ×1 daily
Day 2	Same as the first day, but developed a high-grade fever. Urine output was decreased, and she needed 2 liters of oxygen via nasal cannula	**Full septic workup**: blood culture, urine culture, stool culture, sputum culture, nasal swab, lumbar puncture procalcitonin and CRP. **Serologic tests:** HAV, HCV and HBV, anti-CMV IgM and IgG antibodies. And PCR for multiple viruses	Same as day 1	Piperacillin + Tazobactam 2.25mg x 4 Methylprednisolone 60 mg ×1 daily Famotidine 20 mg ×2 daily Escitalopram 20 mg ×1 daily Rosuvastatin 10 mg ×1 daily Levofloxacin 500 mg every other day Isosorbide mononitrate 20 mg ×2 daily Sevelamer 800 mg ×3 daily
Day 3-6	She upgraded to a 10-liter face mask although her temperature persisted and her urine output decreased.	CMV PCR was sent for external laboratories and preparation for the colonoscopy was completed, but we had to postpone the procedure because of the patient's deteriorating condition.	**Labs**: CMV IgM +ve CMV PCR pending result	Same medications in addition to: Valsartan and Nifedipine 160/30 mg*2 Increased furosemide infusion metolazone 2.5 mg *2 Valagancyclovir orally 450 mg *1
Day 7-8	epigastric pain and right upper quadrant tenderness. In addition, she developed anasarca, and her creatinine was increased.	**PCR**: positive for cmv **Labs**: liver enzymes, amylase and lipase.	Fulminant hepatic failure and pancreatitis (results of labs mentioned on case presentation)	Same medications Albumin 20g x 3 IV ganciclovir 150 mg *2 Discontinuation of: Valsartan and Nifedipine Oral Valganciclovir
Day 9	Vaginal bleeding	**Labs:** coagulating profile	**Labs:** results mentioned on the case presentation showed that patient developed DIC and multi-organ failure	Fresh frozen plasma and packed RBCs were given

## 3 Discussion

Infections remain a major concern in the clinical management of systemic lupus erythematosus (SLE) and are the leading cause of death in SLE patients. Hence, clinicians should be cautious of the possibility of infections in SLE patients, particularly when they present with a fever of unknown origin, which can imitate an SLE flare. Lupus patients are more susceptible to infections due to immune system defects caused by the disease itself or immunosuppressive medications. Immunosuppressive treatment is the most significant predictor of acquired infections in SLE patients, although antimalarial medications appear to have a protective effect. Other indicators of infection include lymphopenia, fever during admission, active renal/mucocutaneous illness, and a hospital stay exceeding seven days (8, 9).

Cytomegalovirus (CMV) is the most common viral infection in these patients. CMV is a double-stranded herpesvirus with more genes than any other herpesvirus, allowing it to evade the host innate and adaptive immune systems (10, 11). The virus carries a lifelong burden of immunological dysfunction and antigenic T cell surveillance. CMV infection is described as evidence of virus replication, regardless of symptoms(not all infected patients experience symptoms), whereas CMV disease refers to symptoms attributed to the infection. In healthy individuals, symptoms may include fever, malaise, mild hepatomegaly, and splenomegaly; however, in immunocompromised individuals, these complications may include acute hepatitis, colitis, pneumonia, encephalitis, iritis, pancreatitis, hemolytic anemia, thrombocytopenia, and retinitis. Diffuse intracellular cytomegalovirus (DIC) is an extremely rare lethal complication of disseminated CMV infection (5–7, 10).

There are very few documented cases of SLE complicated by CMV colitis in the literature. Ikeda et al. (11) reported a case of CMV colitis in a 31-year-old woman with lupus nephritis. In 2016, Berman et al. (3) documented a case of CMV colitis in a patient on belimumab. In 2016, Tachikawa et al. reported a case of an elderly woman with several bleeding colonic ulcers that were removed; unfortunately, the patient did not survive for more than 10 days (12). Takei et al. (13) described a 30-year-old woman with high disease activity SLE, CMV colitis, and *Pneumocystis carinii* pneumonia. Our patient originally presented with gastrointestinal symptoms suggesting colitis and respiratory symptoms suggesting pneumonitis, which proceeded to hepatitis pancreatitis and eventually ended with DIC. These devastating complications were not observed in any of the earlier cases. Cases of DIC due to CMV infection are exceedingly rare (5, 14).

DIC is a rare but potentially fatal manifestation of cytomegalovirus (CMV) disease that has been reported in SLE patients; most reports are case series or single-case descriptions in the context of severe immunosuppression (5). In principle, CMV may lead to endothelial damage and a cytokine-driven hypercoagulable state, which can result in DIC. This condition is often exacerbated by antiphospholipid antibodies and vascular dysfunction associated with SLE. Clinically, consumptive coagulopathy (long PT/aPTT, low fibrinogen, and high D-dimer levels), bleeding, and frequent multiorgan failure are hallmarks of CMV-associated DIC. CMV viremia or tissue-proven infection must be shown, and other potential causes, such as thrombotic microangiopathy or catastrophic antiphospholipid syndrome, must be ruled out. Although mortality in advanced cases is still significant, the recommended course of treatment is to start antiviral therapy (ganciclovir/valganciclovir) as soon as possible and to support the management of DIC-directed therapy as necessary (5, 15).

Pancreatitis is an unusual complication of CMV infection and has only been reported in isolated cases in immunocompromised individuals. Such as those on prolonged steroid or immunosuppressive medication and those with systemic lupus erythematosus. Although rare, it can cause severe symptoms, such as necrotizing pancreatitis; however, the overall incidence is low. CMV should be examined in cases of unexplained pancreatitis, particularly if other common etiologies have been ruled (16, 17),

CMV poses a particular risk to SLE patients because it can interfere with both the differential diagnosis of SLE flare and the use of immunosuppressive medications to treat it. To date, there is limited knowledge on the prevalence of CMV in SLE. In contrast, a comprehensive multicenter retrospective study conducted between April 2000 and May 2005 assessed the prevalence of CMV infection in 7,377 rheumatologic disease patients across eight centers. Most patients had persistent fever, but some also experienced respiratory and gastrointestinal issues. Most patients with CMV antigenemia had received strong immunosuppression over the past 12 months, especially those who had received IV/oral cyclophosphamide, pulse steroids, or high-dose steroids (60 mg–100 mg prednisone) (18). CMV antigenemia was shown to be 35.1% in patients with autoimmune illnesses overall, up to 58.6% in patients with SLE, and 11.4% in individuals with autoimmune diseases other than SLE, according to previous studies (19, 20). The European League Against Rheumatism (EULAR) recommends screening all lupus patients for HIV, HCV, HBV, and CMV infections before starting immunosuppressive therapy, including corticosteroids (21).

Generally, the risk of cytomegalovirus infection in SLE changes with the type and duration of immunosuppressive medication. High-dose corticosteroids (>40 mg prednisone equivalent/day) have been repeatedly linked to CMV reactivation and overt disease, with reported rates of 5%–15% in retrospective cohorts, particularly in the presence of lymphopenia and high disease activity (19). Cyclophosphamide appears to have the most direct link with CMV, with recorded occurrences in 5%–10% of treated individuals, particularly when paired with high-dose steroids. Mycophenolate mofetil is associated with a decreased incidence of CMV (<5%), particularly when used in conjunction with corticosteroids. In contrast, azathioprine has only been associated with sporadic cases of CMV infection in SLE cohorts, suggesting a decreased risk (2, 3, 19, 22).

CMV infection is diagnosed based on high clinical suspicion and laboratory tests. Serologic testing for CMV IgM and IgG antibodies is commonly used in laboratory tests; however, these can be falsely negative. Quantitative polymerase chain reaction (PCR) is used to identify CMV DNA in blood or bodily fluids, provide a more conclusive diagnosis, and aid in determining the viral load and response to treatment. In our case, CMV infection was suspected after excluding any other diagnoses by negative blood, urine, stool, and sputum cultures, nasal swab, and lumbar puncture. Based on her symptoms and immunocompromised state, CMV was suspected. Therefore, IgM was tested and was positive, which revealed active CMV, and PCR detected high viral loads. In our setting, PCR testing was not available and had to be outsourced to external laboratories, resulting in delays in obtaining results, and consequently, a definite diagnosis. Early and precise identification is crucial, as CMV infection in SLE patients can considerably affect the prognosis if not treated promptly (19, 23). Once CMV infection is diagnosed in immunosuppressed patients, the treatment reduction of immunosuppressants is reduced and antiviral drugs such as ganciclovir or valganciclovir are administered (5).

While speculative, the growing use of pulse dosages of methylprednisolone in conjunction with cyclophosphamide may account for the more recent emergence of CMV as an opportunistic infection complicating SLE treatment (3). We recommend screening for CMV using PCR or viral antigenemia in lupus patients who present with symptoms such as fever, diarrhea, and respiratory symptoms, particularly in the context of recent immunosuppressive treatments, and no other infectious organisms have been found. More information is required to decide whether IgG CMV testing should be performed on all SLE patients before aggressive treatment begins and whether it would be safe and beneficial to consider universal prophylaxis or preemptive intervention management for the highest risk patients to lower their risk of contracting this potentially fatal infection.

This study has a few limitations. First, cytomegalovirus (CMV) infection was not recognized promptly, possibly due to a delay in diagnostic tests. Furthermore, the absence of confirmatory tissue collection, such as liver or colon biopsy, further reduces internal validity. Due to the patient’s critical condition, a biopsy was not possible; nonetheless, the diagnosis was confirmed based on compatible clinical characteristics and virologic evidence. Despite these limitations, this case has significant implications for practice. The risk of CMV reactivation is substantial in individuals with systemic lupus erythematosus (SLE) who receive cyclophosphamide and high-dose corticosteroids.

## 4 Conclusion

There are numerous reasons why the implications of this study are noteworthy. Infection is an emerging complication that continues to cause death in SLE patients receiving immunosuppressive therapy. We reported a case of an 18-year-old female with SLE who was infected with CMV following cyclophosphamide therapy, resulting in a fatal disease caused by acute pancreatitis, pneumonia, fulminant liver failure, and colitis, which then progressed to disseminated intravascular coagulation, an extremely unusual complication of CMV. This case report discusses the challenges of disseminated CMV infection in immunocompromised individuals, emphasizing the need for early detection of symptoms and suspicion of infection, encouraging the initiation of antiviral medication, and improving patient prognosis. We present this case to alert clinicians to its devastating implications.

## Data Availability

The original contributions presented in the study are included in the article/supplementary material. Further inquiries can be directed to the corresponding author.
